# The characteristics of gut microbiota composition in patients with depression and relationship with clinical symptoms

**DOI:** 10.3389/fpsyt.2026.1790968

**Published:** 2026-03-11

**Authors:** Ke Hu, Tongtong Zhang, Guoqiang Wang, Kai Zhang

**Affiliations:** 1 College of Mental Health, Bengbu Medical University, Bengbu, Anhui, China; 2 Psychiatry Department of Nangtong Fourth Peopleʼs Hospital, Nantong, Jiangsu, China; 3 Clinical Psychology Department of Wuxi Mental Health Center, Wuxi, Jiangsu, China; 4 Chaohu Hospital of Anhui Medical University, Hefei, Anhui, China

**Keywords:** *Bacteroides*, gut microbiota, *Lachnospiraceae*, major depressive disorder, *Parabacteroides*

## Abstract

**Objective:**

This study was conducted to explore the differences in gut microbiota composition between patients with depressive disorders and healthy individuals, and provide potential biomarkers for the diagnosis of depressive disorders.

**Methods:**

Healthy controls (HC) (n = 31), MDD baseline groups (n = 56), MDD follow-up group were included (n = 22). The composition of gut microbiota was detected by sequencing the V3−V4 region fragments of the 16S rRNA gene in feces. The Hamilton Depression Rating Scale is employed to assess the clinical symptoms of the research subjects. Statistical analyses were conducted using α-diversity test, β-diversity test, t-test, chi-square test, Wilcoxon rank-sum test and Wilcoxon matched-pairs signed-ranks test.

**Results:**

13 differential genera were identified between the HC group and the MDD baseline group, while 6 differential genera were identified in the comparison between the MDD baseline group and follow-up group. Among these, *Bacteroides*, *Parabacteroides*, and *Lachnospiraceae* show promise as potential biomarkers for the diagnosis of MDD.

**Conclusions:**

The findings of this study indicate that there are various gut microbiota communities exhibiting significant differences between healthy individuals and patients with depressive disorders, and it provides valuable insights for further research on the biomarkers for diagnosing depression.

## 1 Introduction

According to the World Health Organization (WHO), Major Depressive Disorder (MDD) affects over 240 million individuals globally. Furthermore, the incidence of MDD increased by more than 25% during the COVID-19 pandemic (1). In 2019, MDD was the second leading cause of healthy life years lost due to disability, accounting for 5.6% of the total across all disabilities (2). However, widely accepted theories regarding the pathogenesis of MDD remain elusive, and first-line treatments derived from these theories alleviate symptoms in only half of patients (3). Thus, there is an urgent need to study the pathogenesis of MDD from new perspectives.

Mounting evidence shows that gut microbiota can affect the host’s brain functions and behaviors via the gut-brain axis (4–6). The gut-brain axis is a bidirectional communication network between the brain and the gastrointestinal tract, primarily involving hypothalamic-pituitary-adrenal (HPA) axis dysfunction, immune response disorders, and other functional system abnormalities. It is emphasized that these functional disorders and abnormalities do not occur independently but result from mutual interactions (7). Gut microbiota plays a key physiological role in maintaining gastrointestinal, hormonal, immune, and neural homeostasis, and its imbalance may induce MDD (8). In our previous study, we established a rat model of learned helplessness (LH) to compare changes in intestinal flora before and after the onset of depression. The results showed that the abundance of intestinal flora in depressed rats was lower than in healthy controls (9). Other researchers using animal models of depression have also reported that gut microbiota can induce depression-like behaviors by regulating host metabolism (10). In addition to animal models, previous studies have found that the diversity of gut microbiota is decreased in patients with MDD compared with healthy individuals (11). Our study also identified differences in the structural composition of gut microbiota between MDD patients and healthy people; the relative abundances of 28 genera and 40 species were significantly different at the genus and species levels, respectively (12). These findings suggest that further exploration of the role of gut microbiota in the onset of MDD may help to reveal its pathogenesis.

Clinically, depressive symptoms are complex, involving cognition, motivation, emotion, behavior, and other domains, often with low specificity. Traditional assessment relies on clinician-led interviews, with clinical inferences drawn based on evaluation scores. However, as patient reports can be variable, this approach may lead to misdiagnosis and inappropriate treatment (13). Furthermore, patients with severe MDD already present a high risk of suicide or self-harm. Therefore, identifying novel biomarkers to distinguish MDD patients by severity through gut microbiota studies is of significant clinical importance. Given the close relationship between MDD and gut microbiota, we conducted this study to analyze differences in gut microbiota between MDD patients and healthy controls. By including a follow-up with a subset of patients, we aimed to identify potential microbial markers associated with MDD severity.

## 2 Methods

### 2.1 Participants

The participants in this study were recruited from the Wuxi Mental Health Center and surrounding communities. The study protocol was reviewed and approved by the Ethics Committee of the Wuxi Mental Health Center. All participants were fully informed about the study and provided written informed consent. Each patient met the diagnostic criteria for Major Depressive Disorder (MDD) as defined by the Diagnostic and Statistical Manual of Mental Disorders, 5th Edition (DSM-5). The severity of MDD was assessed using the 24-item Hamilton Depression Rating Scale (HAMD-24) (14). A total of 56 patients with MDD (23 with first-episode and 33 with recurrent depressive disorder) who met the inclusion criteria were enrolled. This distribution allowed us to explore potential medication-related effects on gut microbiota in subsequent analyses. A follow-up study was conducted one month after enrollment. However, due to the impact of the COVID-19 pandemic, we successfully collected follow-up data from 22 patients in the depression group (7 with first-episode and 15 with recurrent depressive disorder). Additionally, 31 healthy individuals were recruited as a control group.

The exclusion criteria for this study were as follows: (1) presence of comorbid psychiatric disorders; (2) history of organic brain lesions or severe traumatic brain injury; (3) significant cardiovascular, hepatic, or renal dysfunction, metabolic disorders, or other serious systemic diseases; (4) pregnancy or lactation; (5) use of antibiotics or microbial ecology modulators within the past month; (6) abnormal laboratory test results (e.g., liver function tests, complete blood count, urinalysis); (7) participation in another clinical trial within the past three months; and (8) severe suicidal ideation. These exclusion criteria were specifically designed to minimize the impact of potential confounders, including antibiotics, probiotics, and comorbid medical conditions.

Age, body mass index (BMI), and sex ratio were matched across the three groups. The detailed demographic and clinical characteristics of the participants are presented in **Table 1**.

**Table 1:** Characteristics of the included subjects.

Basic information	HC	MDD baseline	*p*-value^a^(*t/χ^2^*)	MDD follow-up	*p*-value^b^(*t/χ^2^*)
Number	31	56	*-*	22	*-*
Gender, male/female	17/14	21/35	0.12(2.44)	8/14	0.93(0.01)
Age, years	31.32(11.51)	31.13(10.51)	0.94(0.60)	32.64(10.23)	0.57(0.01)
BMI	23.02(2.10)	23.34(1.82)	0.45(0.65)	23.31(1.81)	0.94(0.01)
HAMD	0.65(0.66)	26.43(9.73)	<0.001(36.92)	12.23(10.76)	<0.001(2.08)
Medication	0	39	*-*	22	*-*

Chi-square test and t-test were used for the comparison of differences between groups; *p*-value^a^ was from HC vs. MDD baseline; *p*-value^b^ was from MDD baseline vs. MDD follow-up; HC, healthy controls; MDD, major depressive disorder; BMI, body mass index; HAMD, Hamilton Depression Scale.

### 2.2 Assessment and stool collection

General information from outpatients and inpatients with MDD was collected, and their depressive symptoms were assessed using the HAMD-24 scale. The assessments were conducted by two trained psychiatrists to ensure standardization, achieving an inter-rater reliability with an intraclass correlation coefficient (ICC) of 0.86. Stool samples were collected within 24 hours after the assessment. Participants were instructed to empty their bladders prior to sample collection to minimize potential bacterial contamination. Approximately 2 grams of fecal matter from the internal part of the stool, which had not been exposed to air or contacted the ground, was collected using a sterile spoon. The sample was then placed into a disposable sterile tube and immediately stored at -80 °C. Data and fecal samples from the control group were collected following the same protocol.

### 2.3 DNA extraction and PCR amplification

After adding 0.5 g sample, the total DNA of the microbial community was extracted by DNA extraction kit, and the quality of the extracted DNA was detected by 1% agarose gel electrophoresis. The V3-V4 hypervariable region of the bacterial 16S rRNA gene was amplified with primer pairs 338F (5’-ACTCCTACGGGAGGCAGCAG-3’) and 806R (5’-GGACTACHVGGGTWTCTAAT-3’) using an ABI GeneAmp^®^ 9700 PCR thermocycler (ABI, CA, USA). The PCR reaction system was as follows: 5×TransStart FastPfu buffer 4 μL, dNTPs (2.5 mmol/L) 2 μL, upstream and downstream primers (5 μmol/L) 0.8 μL each, TransStart FastPfu DNA polymerase 0.4 μL. 10 ng of template DNA was supplemented to 20 μL. Three replicates were performed for each sample. PCR reaction conditions: 95 °C for 3 min; 27 cycles of 95 °C for 30 s, 55 °C for 30 s, and 72 °C for 30 s. 72 °C for 10 min and finally stored at 4 °C.

### 2.4 Illumina MiSeq sequencing

Purified amplicons were pooled in equimolar and paired-end sequenced on Illumina MiSeq PE300 platforms (2 × 300 bp) according to the standard protocols by Majorbio Bio-Pharm Technology Co., Ltd. (Shanghai, China). The 468 bp amplicons of the V3-V4 region were fully covered by both sequencing platforms.

### 2.5 Processing of sequencing data

The raw 16S rRNA gene sequencing reads were demultiplexed, quality-filtered by fastp version 0.20.0 (15) and merged by FLASH version 1.2.7 (16). Operational taxonomic units (OTUs) were clustered at 97% similarity cutoff using UPARSE version 7.1 (17), and chimeric sequences were identified and removed. ASV methods such as DADA2 were not employed in this study. The taxonomy of each OTU representative sequence was analyzed by RDP Classifier version 2.2 (18) against the SILVA 16S rRNA database (version 138) using a confidence threshold of 0.7. Relative abundances were calculated by dividing the number of reads assigned to each taxon by the total number of reads per sample. No additional normalization (e.g., rarefaction) was applied. All samples were processed in a single batch using the same DNA extraction, PCR amplification, and sequencing protocols to minimize batch effects.

### 2.6 Metagenome data analysis

#### 2.6.1 α diversity analysis

Alpha (α) diversity, reflecting the abundance and diversity of microbial communities within samples, was assessed using several diversity indices. Differences in α diversity indices between groups were analyzed using the Student’s t-test. Specifically, community richness was evaluated with the Chao1 index, while community diversity was assessed using the Shannon and Simpson indices. The coverage index was employed to evaluate sequencing depth.

#### 2.6.2 β diversity analysis

Beta (β) diversity was analyzed to assess the differences in microbial community composition between groups. Principal co-ordinates analysis (PCoA) based on Bray-Curtis distances was employed to visualize the similarity or dissimilarity of community structures. To statistically test for differences in community composition, Analysis of Similarities(ANOSIM) was performed with 999 permutations. ANOSIM is a widely accepted non-parametric method for β-diversity comparison in microbiome studies, generating an R-value (effect size) and *p*-value (significance). The R-value ranges from -1 to 1, with positive values indicating greater between-group dissimilarity than within-group dissimilarity. The *p*-value <0.05 indicates a significant difference in community structure between the two compared groups.

#### 2.6.3 Community composition analysis

Bar charts were used to visualize the microbial community composition and the relative abundances of microorganisms across all groups. This presentation allows for a clear observation of the changing trends in species distribution between groups, such as controls and treatments, and serves as a primary method for understanding the overall community structure within samples.

#### 2.6.4 Multilevel species difference discriminant analysis

Multilevel species difference discriminant analysis was performed to identify differentially abundant species across multiple taxonomic hierarchies. LEfSe (Linear Discriminant Analysis Effect Size) was employed to identify taxa with the largest effect sizes discriminating between groups. LEfSe is presented as a complementary visualization tool illustrating effect sizes, while formal statistical inference is based on the more conservative FDR-corrected Wilcoxon tests (**Tables 2**, **3**). All taxa identified by LEfSe were cross-validated with these FDR-corrected analyses. Genera that were significant in both the Wilcoxon rank-sum test and showed high LDA scores were considered our core focus.

**Table 2:** Significance test of differences between groups at the genus, HC VS. MDD T0.

Genus	HC-Mean (%)	HC-*Sd* (%)	MDD T0-Mean (%)	*MDD* T0-*Sd* (%)	Lower ci	Upper ci	FDR *q*-value	Effect size
*Bacteroides*	2.347	4.507	6.978	9.619	1.892	7.370	0.001^**^	4.632
*Romboutsia*	1.275	1.585	0.778	1.427	-0.982	-0.012	0.002^**^	-0.497
*Parabacteroides*	0.887	3.190	0.425	0.623	-1.312	0.388	0.008^**^	-0.462
*Holdemanella*	0.520	0.901	0.143	0.433	-0.658	-0.096	0.003^**^	-0.377
*Eggerthella*	0.048	0.121	0.196	0.351	0.051	0.245	0.001^**^	0.148
*Akkermansia*	0.036	0.045	0.015	0.020	-0.039	-0.003	0.022^*^	-0.022
*UBA1819*	0.030	0.058	0.260	0.698	0.088	0.372	0.001^**^	0.230
*Coriobacteriales*	0.014	0.042	0.004	0.026	-0.026	0.006	0.004^**^	-0.010
*Erysipelatoclostridium*	0.010	0.040	0.061	0.117	0.016	0.086	0.001^**^	0.051
*Clostridium_innocuum_group*	0.009	0.027	0.038	0.081	0.007	0.051	0.001^**^	0.029
*norank_f:Prevotellaceae*	0.007	0.022	0.002	0.011	-0.014	0.004	0.018^*^	-0.005
*Chryseobacterium*	0.004	0.021	0.001	0.000	-0.012	0.004	0.005^**^	-0.003
*Holdemania*	0.003	0.003	0.010	0.012	0.004	0.010	0.001^**^	0.007

Wilcoxon rank-sum test was used to compare the differences between the two groups, the *q*-value has been corrected by FDR; q^*^<0.05, q^**^<0.01, The *p*-value has been corrected by FDR; Mean(%) was the average relative abundance of the bacterial genus, and *Sd* (%) was the standard deviation; Lower ci and Upper ci represent the lower and upper limits of the confidence interval, respectively; The effect size greater than 0 indicates that the relative abundance of this bacterial genus in the MDD baseline group is higher than that in the HC group. HC, healthy controls, MDD T0, MDD baseline.

**Table 3:** Significance test of differences between groups at the genus, MDD T0 VS. MDD T1.

Genus	MDD T0-Mean (%)	MDD T0-*Sd* (%)	MDD T1-Mean (%)	*MDD* T1*-Sd* (%)	Lower ci	Upper ci	FDR *q*-value	Effect size
*Lachnospiraceae*	0.028	0.028	0.066	0.060	0.010	0.066	0.004^**^	-0.037
*Parabacteroides*	0.552	0.556	0.246	0.269	-0.568	-0.044	0.004^**^	0.306
*Lachnoclostridium*	0.881	0.708	0.581	0.803	-0.700	-0.100	0.010^*^	0.300
*Flavonifractor*	0.127	0.191	0.076	0.157	-0.131	-0.019	0.017^*^	0.052
*Ruminococcus_gnavus_group*	1.961	2.951	0.593	1.028	-2.348	-0.388	0.023^*^	1.368
*Eubacterium_hallii_group*	3.422	3.128	5.062	4.183	-0.245	3.525	0.040^*^	-1.640

Wilcoxon matched-pairs signed-ranks test was used to compare the differences between the two groups, the *q*-value has been corrected by FDR; q^*^<0.05, q^**^<0.01; Mean(%) was the average relative abundance of the bacterial genus, and *Sd* (%) was the standard deviation; Lower ci and Upper ci represent the lower and upper limits of the confidence interval, respectively; The effect size greater than 0 indicates that the relative abundance of this bacterial genus in the MDD baseline group is higher than that in the MDD follow-up group. MDD T0, MDD baseline; MDD T1, MDD follow-up.

### 2.7 Statistical analysis

Student’s t test and chi-square test were used to analysis whether there were significant differences in demographic data. In the differential analysis, To reduce the risk of false-positive findings caused by low-abundance genera, we excluded genera with mean relative abundance <0.001% in both comparison groups. For all remaining genera, raw *p*-values were calculated using the Wilcoxon rank-sum test for the comparison between HC and MDD baselin group, and the Wilcoxon matched-pairs signed-ranks test for the comparison between MDD baselin and MDD follow-up group. In addition, we applied the Benjamini-Hochberg false discovery rate (FDR) procedure, and FDR-corrected *q*-values < 0.05 were considered statistically significant.

## 3 Results

### 3.1 Comparison of gut microbiota diversity

The results of the α-diversity analysis are summarized in **Table 4**. The Chao1, Coverage, Shannon, and Simpson indices were employed to assess microbial community richness and diversity. The coverage indices for all three groups were approximately 1, indicating that the sequencing depth was sufficient, with coverage exceeding 99% for all samples, which confirms the reliability of the sequencing data. No statistically significant differences were found in any of the α-diversity indices between the HC group and the MDD baseline group, or between the MDD baseline group and the MDD follow-up group.

**Table 4:** α diversity test for differences between groups.

Estimators	HC		MDD baseline		MDD follow-up		*p*-value^a^	*p*-value^b^
	Mean	*SD*	Mean	*SD*	Mean	*SD*		
chao	345.28	105.05	314.62	75.89	74.14	0.68	0.06	0.68
coverage	1.00	0.00	1.00	0.00	1.00	0.50	0.87	0.68
shannon	3.20	0.76	3.25	0.54	0.63	0.60	0.64	0.68
simpson	0.11	0.12	0.10	0.07	0.11	0.60	0.88	0.68

t-test was used to compare the differences in index values between groups; *p*-value^a^ was from HC vs. MDD baseline; *p*-value^b^ was from MDD baseline vs. MDD follow-up; HC, healthy controls.

**Figure 1:**
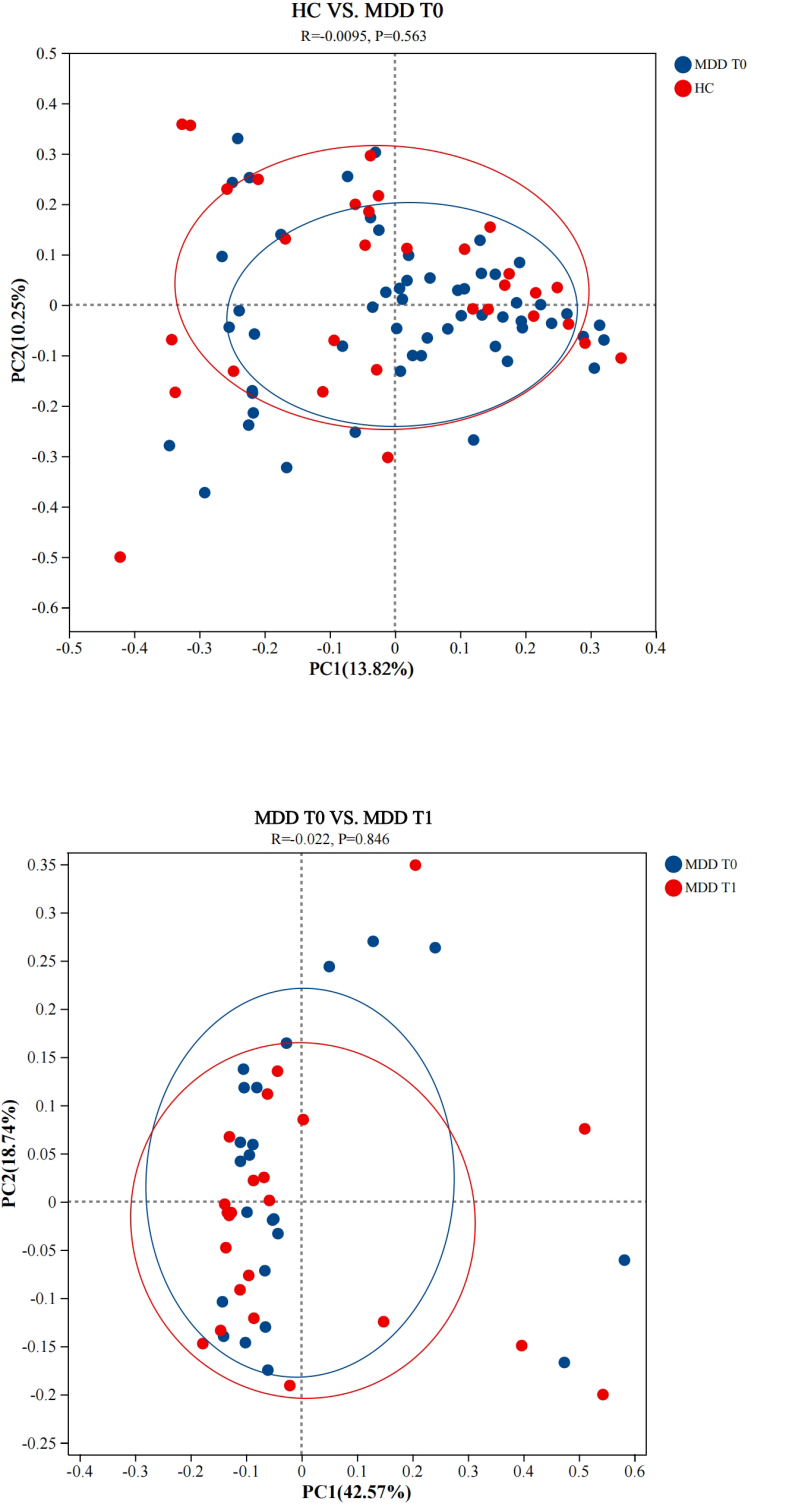
Principal co-ordinates analysis at the genus level. HC, healthy controls. MDD T0, MDD baseline; MDD T1, MDD follow-up. ANOSIM confirmed no significant separation between HC and MDD T0 (R = 0.0095, p = 0.563) or between MDD T0 and MDD T1 (R = -0.022, p = 0.846). p-value > 0.05 indicates no significant difference in community structure between the two compared groups.

In the β-diversity analysis, the similarity and differences in gut microbiota communities between groups were visualized using PCoA based on Bray-Curtis distances. As shown in **Figure 1**, the PCoA plot revealed that the sample points from the HC group, the MDD baseline group, and the MDD follow-up group were closely clustered. To statistically evaluate group separation, ANOSIM was performed with 999 permutations. The results confirmed no significant differences in overall community structure: HC vs. MDD T0 (R = 0.0095, *p* = 0.563) and MDD T0 vs. MDD T1 (R = -0.022, *p* = 0.846). The R-values close to zero and *p*-value > 0.05 indicate that the overall composition of gut microbiota is similar among these groups, consistent with the visual observation from the PCoA plot.

### 3.2 Compositional analysis of gut microbiota

**Figure 2:**
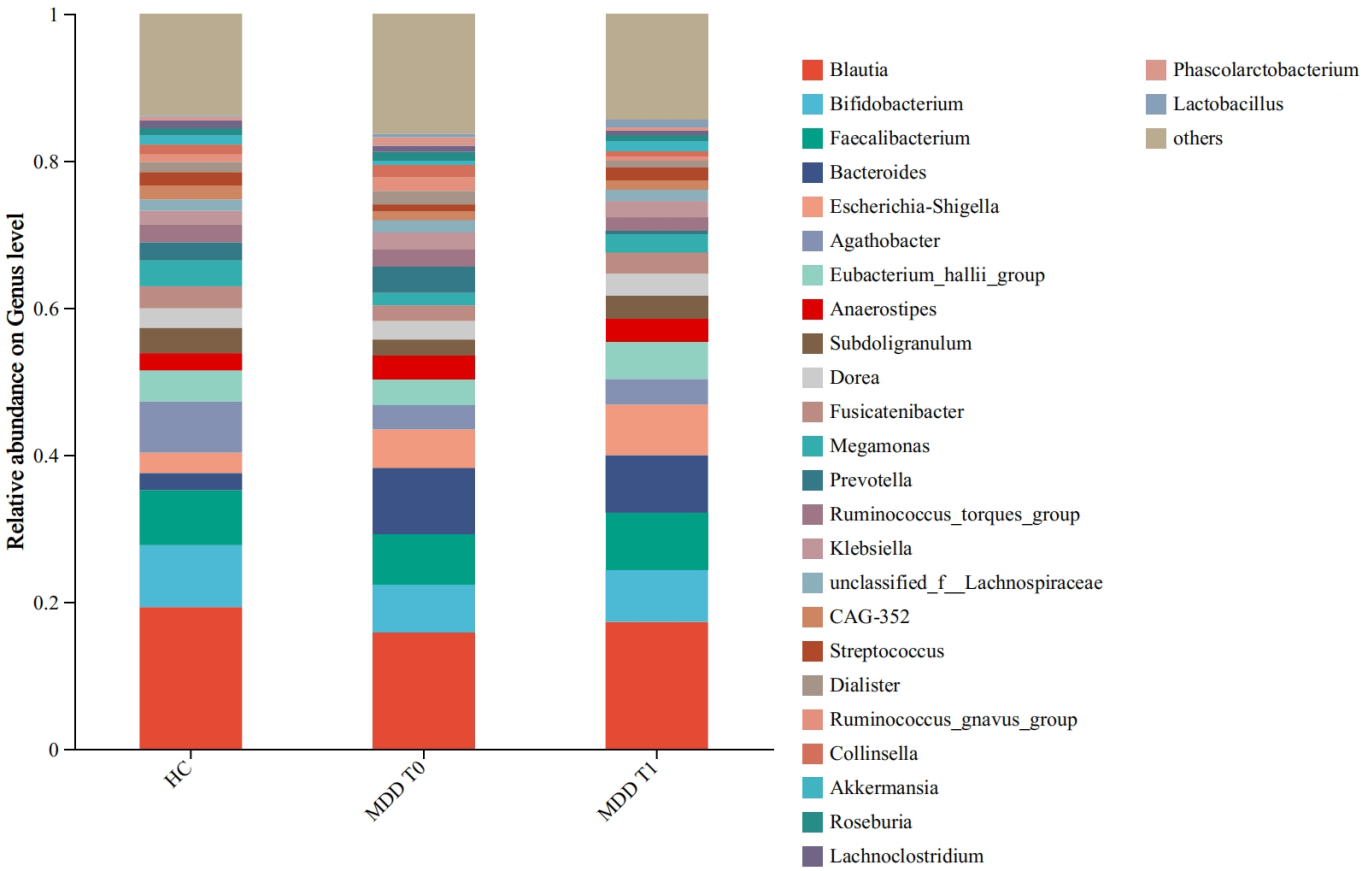
Community barplot analysis at the genus level. HC, healthy controls. MDD T0, MDD baseline; MDD T1, MDD follow-up. This figure is descriptive, illustrating the overall composition of the gut microbiota. For statistical comparisons with FDR-corrected q-values and effect sizes, see **Tables 2** and **3**.

The compositional analysis of gut microbiota across the different groups is illustrated in **Figure 2**. **Figure 2** shows the predominant bacterial genera, revealing that *Blautia, Bifidobacterium*, and *Faecalibacterium* were consistently highly abundant across all three groups. The relative abundances of *Bacteroides* were higher in MDD patients, whereas *Agathobacter* was more abundant in healthy controls. Further statistical analysis is required to confirm the significance of these observed intergroup differences.

### 3.3 Analysis of the differences in the relative abundance of bacterial genera

We identified bacterial genera with significant differences in relative abundance between the HC group and the MDD baseline group, as well as between the MDD baseline and MDD follow-up groups. After excluding genera with extremely low abundance (<0.001%) and applying FDR-corrected Wilcoxon rank-sum test, 13 bacterial genera were identified as significantly different between the HC and MDD baseline groups (*q* < 0.05; **Table 2**). The most robust differences were observed for *Bacteroides* (*q* = 0.001, effect size = 4.632), which was enriched in MDD patients, and *Parabacteroides* (*q* = 0.008, effect size = -0.462), which was depleted in MDD patients. In the 22 participants with complete follow-up data, paired Wilcoxon signed-ranks test with FDR correction identified 6 genera with significant changes from baseline to one-month follow-up. as detailed in **Table 3**. Notably, the genera *Parabacteroides* exhibited significant differences in both comparisons. The relative abundance of *Parabacteroides* was highest in the HC group, intermediate in the MDD baseline group, and lowest in the MDD follow-up group, showing a consistent decreasing trend.

### 3.4 Multilevel species difference discriminant analysis

The LEfSe method was employed to identify microbial taxa with significant differences across multiple taxonomic levels (from phylum to species) and to estimate their effect sizes. The LDA score quantifies the degree to which each taxon’s abundance contributes to the observed differences between groups; a higher score indicates a greater influence.

**Figure 3:**
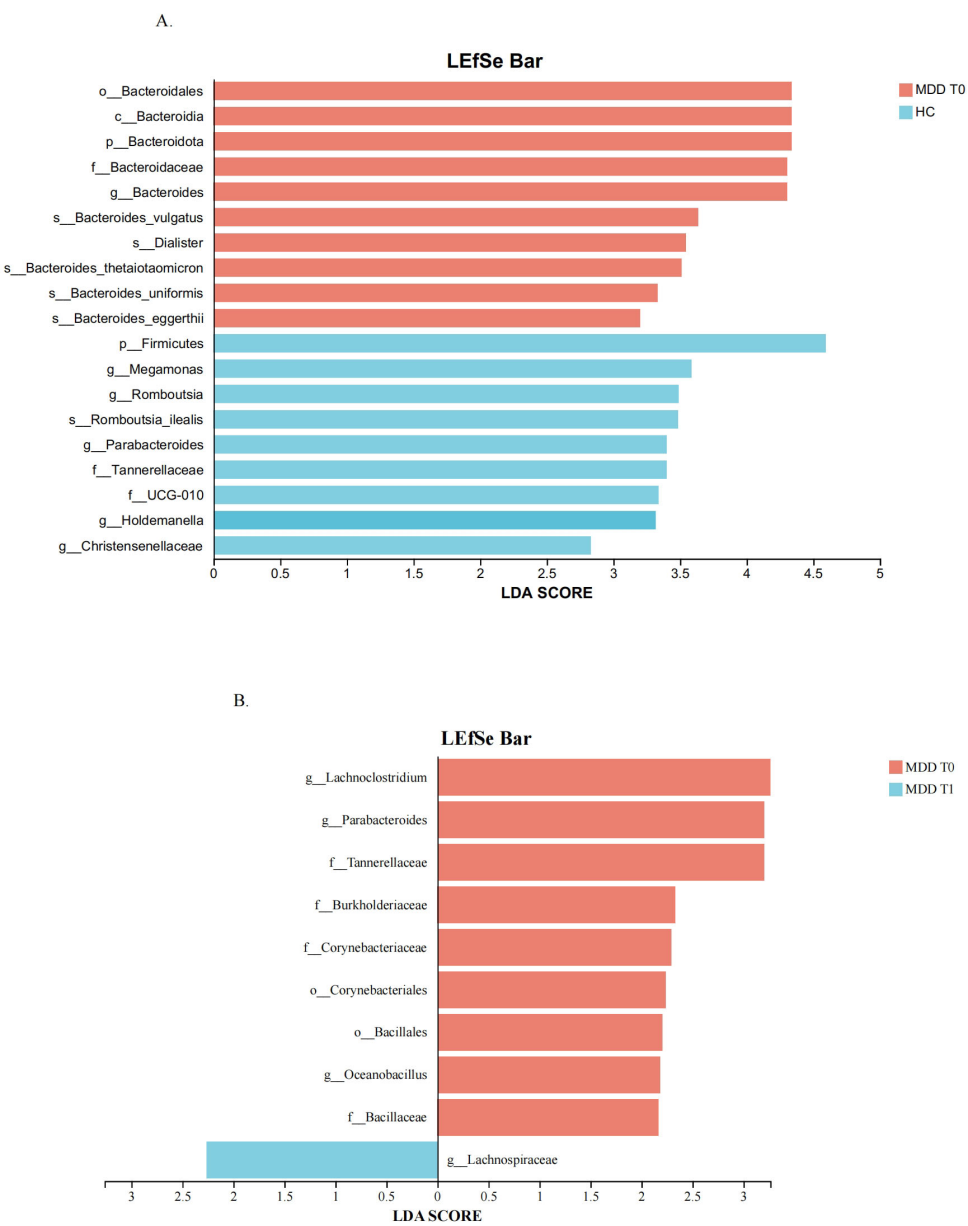
**(A)** Differentially enriched gut microbiota of HC and the MDD T0. **(B)** Differentially enriched gut microbiota of MDD T0 and the MDD T1. HC, healthy controls. MDD T0, MDD baseline; MDD T1, MDD follow-up.

In the comparison between the HC and MDD baseline groups, various species within the genus *Bacteroides* were identified as the most significant biomarkers, exhibiting higher abundance in MDD patients and thus driving the divergence in gut microbiota composition. Furthermore, the phylum *Firmicutes* was significantly enriched in healthy controls and also constituted a major differentiating taxon based on its LDA score. These results are presented in **Figure 3A**. In the comparison between the MDD baseline and follow-up groups, taxa such as *Lachnoclostridium, Parabacteroides, Oceanobacillus, Bacillaceae* et. were more enriched in the baseline group. In contrast, only *Lachnospiraceae* showed an increase in the follow-up group, as detailed in **Figure 3B**. Among these, *Bacteroides, Parabacteroides*, and *Lachnospiraceae* also showed significant differences in the Wilcoxon rank-sum test, making them core subjects of interest in this study.

### 3.5 Comparison between first-episode and recurrent MDD patients

To directly assess whether medication exposure might confound our findings, we performed an additional analysis comparing first-episode (minimally treated, n=23) and recurrent (previously treated, n=33) patients within the MDD baseline group. Among the 23 patients with first-episode MDD, 17 were not taking medication at the time of data collection. For the remaining 6 patients, data were collected within three days of their initial presentation at the outpatient clinic or ward, thereby minimizing the potential confounding effects of medication. The results are presented in **Table 5**. After FDR correction, 4 genera showed significant differences (*q* < 0.05): *Oceanobacillus, Turicibacter, Kroppenstedtia, and unclassified_f:Peptostreptococcaceae*. These represent taxa that may be influenced by antidepressant treatment. Crucially, none of the key genera identified in our primary HC vs. MDD comparison—including *Bacteroides, Parabacteroides*, showed significant differences between first-episode and recurrent patients after FDR correction. This evidence that the core MDD-associated microbial alterations are largely independent of medication exposure, while a distinct set of taxa may be sensitive to antidepressant treatment.

**Table 5:** Significance test of differences between groups at the genus, First-episode MDD VS. Recurrent MDD.

Genus	First-episode Mean(%)	First-episode *Sd*(%)	Recurrent -Mean(%)	Recurrent- *Sd*(%)	Lower ci	Upper ci	FDR *q*-value	Effect size
*Oceanobacillus*	0.001	0.001	0.003	0.003	-0.003	-0.001	0.028^*^	-0.002
*Turicibacter*	0.142	0.345	0.049	0.153	0.034	0.265	0.031^*^	0.093
*Kroppenstedtia*	0.000	0.001	0.002	0.002	-0.003	-0.001	0.049^*^	-0.002
*unclassified_f:Peptostreptococcaceae*	0.022	0.025	0.009	0.015	0.003	0.024	0.041^*^	0.013

Wilcoxon rank-sum test was used to compare the differences between the two groups, *q*-value has been corrected by FDR; q^*^<0.05, q^**^<0.01; Mean(%) was the average relative abundance of the bacterial genus, and *Sd* (%) was the standard deviation; Lower ci and Upper ci represent the lower and upper limits of the confidence interval, respectively; The effect size greater than 0 indicates that the relative abundance of this bacterial genus in the Recurrent MDD group is higher than that in the First-episode MDD group.

## 4 Discussion

This study was designed to identify gut microbial biomarkers for the differentiation and diagnosis of depressive disorders by comparing HC to patients with MDD, as well as by examining MDD patients before and after treatment. The results revealed no significant differences in overall microbial diversity among the groups. However, comparative analysis identified 13 differentially abundant genera between the HC group and the MDD baseline group, and 6 differentially abundant genera between the MDD baseline and follow-up groups. Notably, genera such as *Bacteroides, Parabacteroides* and *Lachnospiraceae* consistently emerged across multiple analyses, suggesting their potential as diagnostic biomarkers for MDD.

In the α-diversity analysis of this study, no statistically significant differences were observed in any of the indices between the HC and MDD groups (*p* > 0.05). Previous research has revealed a significant correlation between gut microbiota diversity and depressive disorders, with a general consensus indicating reduced microbial abundance and diversity in individuals with depression (19, 20). However, an increasing body of evidence presents divergent conclusions. For instance, Shen et al. observed that MDD patients treated with SSRIs exhibited significantly higher α-diversity compared to both a follow-up group and a healthy control group (21). Similarly, another study found a significant reduction in the Chao1 index within an MDD group, yet paradoxically, no significant differences were observed using the Shannon or inverse Simpson’s indices (4). Current research findings therefore remain inconclusive regarding the utility of gut microbiota diversity as a reliable marker to distinguish MDD individuals from healthy populations, necessitating further investigation. This inconsistency may stem from multiple factors, including variations in participant selection criteria. For example, one study restricted participants to ages 18–65 with HAMD scores exceeding 24 (21), whereas our study encompassed participants aged 20–75 with HAMD scores above 18. Additional confounding factors likely involve differences in ethnic backgrounds, dietary habits, and fecal sampling protocols, all of which can significantly influence gut microbiota composition. Consequently, establishing standardized criteria for participant enrollment and matching, alongside comprehensive guidelines for sampling and detection, is crucial for advancing this field of research.

The community bar plots revealed variations in the abundance of specific microbial taxa across groups, allowing for the observation of dominant communities. Integrated with the significance testing results (*Bacteroides* in HC VS. MDD T0, *p* < 0.05), *Bacteroides* emerged as potential biomarkers for distinguishing MDD patients from healthy controls. The genus *Bacteroides* is among the bacterial taxa most frequently associated with depressive disorders. Consistent with our findings, numerous studies have reported an increased abundance of *Bacteroides* as a significant feature of MDD (22, 23). Based on currently available evidence, *Bacteroides*—a genus recognized for its pro-inflammatory capacity—appears to influence the pathogenesis and progression of psychiatric disorders in the host through a network of interconnected biological pathways. Evidence suggests that *Bacteroides* influences the onset and progression of mental disorders primarily by modulating host inflammatory and immune responses. Furthermore, lipopolysaccharide (LPS), a key component of the outer membrane of Gram-negative bacteria such as *Bacteroides*, can translocate into the central nervous system when intestinal barrier integrity is compromised, thereby inducing systemic low-grade inflammation (24). A recent study on microbiota-cytokine correlations further reported a positive association between the relative abundance of *Bacteroides* and pro-inflammatory cytokines, including IL-1β, IFN-γ, IL-6, and TNF-α (25). Building upon this understanding, the following sequence of events is posited to occur: intestinal microbiota dysbiosis elevates the expression of mucosal inflammatory markers, including NLRP3 and NF-κB (26, 27). This upregulation compromises the integrity of the intestinal barrier, leading to increased mucosal permeability. Consequently, pro-inflammatory bacteria such as Bacteroides—along with their immunogenic byproducts, notably lipopolysaccharide (LPS)—translocate into the systemic circulation, thereby instigating a systemic immune response. This cascade is marked by elevated levels of peripheral pro-inflammatory cytokines, including interleukin-1β (IL-1β) and interferon-gamma (IFN-γ). These mediators can access the brain either through circumventricular routes or via direct neural pathways, where they precipitate neuroinflammation. Such neuroinflammatory processes are known to disrupt the delicate homeostasis of neurotransmitter systems, impairing the synthesis, release, and metabolism of key monoamines (28). The resultant decline in neurotransmitters such as serotonin and dopamine ultimately compromises neural function, manifesting clinically as depressive symptomatology. This signaling cascade is proposed as a core functional mechanism within the microbiota–metabolite–inflammation axis.

The statistical analysis of microbial differences identified *Parabacteroides* as genera that varied significantly in both the HC vs. baseline MDD and the follow-up vs. baseline MDD comparisons. *Parabacteroides* is increasingly recognized as a promising candidate for next-generation beneficial microorganisms. Accumulating evidence has demonstrated that its abundance is significantly altered in patients with depression at the genus level (29). For instance, Deng et al. showed that alleviating anxiety and depression-like behaviors in mice was associated with a reduced abundance of *Parabacteroides* and enhanced tryptophan biosynthesis (30). As an important member of the intestinal microbiota, *Parabacteroides* plays a significant role in host metabolic functions and immune regulation (31). Although the current understanding of its role in health regulation remains incomplete, further investigation of its function within the gut-brain axis is essential to unlock its therapeutic potential for the precise diagnosis and treatment of MDD.

The multilevel species difference discriminant analysis further identified *Bacteroides* as a potential keystone species contributing to the gut microbiota disparities between healthy individuals and patients with MDD. In the LEfSe comparison between the baseline and follow-up groups, *Lachnospiraceae* was the sole bacterial family exhibiting a significantly higher relative abundance in the follow-up cohort. Many members of *Lachnospiraceae* can metabolize complex polysaccharides into short-chain fatty acids (SCFAs), Mechanistically, SCFAs exert their antidepressant effects through multiple interconnected pathways. First, they preserve intestinal barrier integrity by upregulating tight junction proteins, including Claudin-1, ZO-1, and Occludin, thereby preventing the translocation of pro-inflammatory lipopolysaccharide (LPS) into the systemic circulation (32). Second, SCFAs exert anti-inflammatory effects by binding to free fatty acid receptor 3 (FFAR3), which inhibits microglial M1 polarization, reduces the production of pro-inflammatory cytokines (IL-6, TNF-α), and enhances secretion of the anti-inflammatory cytokine IL-10 (33). Third, SCFAs stimulate the release of serotonin (5-HT) from enterochromaffin cells, influencing mood regulation via the gut–brain axis (32). Fourth, an animal study suggests that the gut microbiota can influence central nervous system serotonin (5-HT) synthesis through SCFA-mediated modulation of tryptophan metabolism, thereby exerting a significant impact on the pathogenesis and progression of depression (34). Collectively, these findings suggest that anti-inflammatory activity represents a key mechanistic pathway through which SCFAs exert their antidepressant effects. This framework offers a plausible explanation for the clinical observations in the present study, wherein symptomatic improvement was accompanied by a concomitant increase in the relative abundance of *Lachnospiraceae*. Conversely, the elevated levels of *Bacteroides* observed in individuals with depression point to a potential dysbiotic shift. These two bacterial taxa may thus play opposing roles in the pathophysiology of depression, with *Lachnospiraceae* potentially contributing to remission via SCFA-mediated anti-inflammatory pathways, while *Bacteroides* may be associated with a pro-inflammatory state that characterizes the active phase of the disorder.

From a clinical translation perspective, emerging evidence suggests that baseline gut microbiota composition may serve as a predictor of antidepressant treatment response. Through systematic review and quantitative analysis, researchers have conceptualized two distinct microbial functional categories: “Microholders,” defined as beneficial bacteria that increase following antidepressant treatment and are typically associated with anti-inflammatory properties, short-chain fatty acid production, and restoration of a eubiotic microbial state; and “Microlencers,” characterized as harmful taxa that decrease after antidepressant intervention and are generally linked to pro-inflammatory activity, pathogenic potential, or depression-associated dysbiosis. Assessing the baseline abundance of these microbial populations may offer predictive value for treatment efficacy, while strategically modulating the Microholder/Microlencer ratio could potentially enhance therapeutic outcomes (35). Although these findings remain preliminary, they point toward the emerging clinical utility of gut microbiota profiling as a biomarker for treatment guidance and personalized psychiatric care. Future studies incorporating fecal microbiota transplantation from our MDD patients into germ-free or antibiotic-treated mice would help establish causal relationships between the specific taxa identified here (e.g., *Bacteroides, Parabacteroides, Lachnospiraceae*) and depressive-like behaviors. Such animal experiments could also elucidate the mechanistic pathways—such as SCFA-mediated anti-inflammatory effects or LPS-induced neuroinflammation—that underlie the associations observed in our human cohort, thereby strengthening the translational evidence base.

Unexpectedly, although patients in the follow-up group showed significant symptomatic improvement, the abundances of bacteria such as *Bacteroides* did not revert to the levels observed in healthy controls. This discrepancy could be attributed to the relatively short follow-up period, which may have been insufficient for full microbial recovery. These observations highlight the need for further refinement in research methodologies and participant recruitment procedures.

Several limitations of this study should be acknowledged. First, the sample size was limited, especially within the follow- up cohort. Second, while major confounders such as age, body mass index, and sex were controlled through inclusion criteria, we did not collect detailed information on dietary habits or smoking status—factors known to influence gut microbiota composition—due to logistical constraints during the COVID-19 pandemic. Third, the impact of medication on gut microbiota composition cannot be overlooked. Our classification of first-episode vs. recurrent patients serves only as a proxy for medication exposure and does not capture specific antidepressant classes, dosages, or treatment durations. Fourth, technical constraints related to the expiration of our cloud-based bioinformatics platform license prevented additional multivariable modeling linking microbiota to HAMD severity scores. Future studies should incorporate standardized dietary assessments, smoking history, and detailed medication logs to enable more comprehensive confounder control.

## 5 Conclusion

In conclusion, this study demonstrates distinct compositional differences in gut microbiota between healthy individuals and patients with major depressive disorder. Specifically, 13 differentially abundant genera were identified between the HC and MDD baseline groups, and 6 genera differed significantly between the MDD baseline and follow-up groups. Notably, *Bacteroides, Parabacteroides*, and *Lachnospiraceae* emerged as promising microbial biomarkers for MDD diagnosis. These findings provide a valuable foundation for further elucidating the role of gut microbiota in the pathogenesis of depression and for developing novel diagnostic strategies.

## Data Availability

The raw data supporting the conclusions of this article will be made available by the authors, without undue reservation.
